# Tissue-specific mitochondrial DNA, *MT-TF,* pathogenic variants in mitochondrial myopathies

**DOI:** 10.1016/j.ymgmr.2025.101230

**Published:** 2025-05-27

**Authors:** Sylvia Rose, Aurélien Trimouille, Didier Lacombe, Edoardo Malfatti, Zahra Assouline, Julie Steffann, Isabelle Desguerre, Arnold Munnich, Agnès Rötig, Giulia Barcia

**Affiliations:** a Université Paris Cité, Institut Imagine, Génétique des maladies mitochondriales, INSERM UMR1163, Paris, France; b Service de Médecine Génomique des Maladies Rares, Hôpital Necker Enfants Malades, Paris, France; c Service de Génétique Médicale, CHU de Bordeaux-GH Pellegrin, Bordeaux, France; d Service d'Histologie Département de Pathologie, Hôpital Universitaire Henri Mondor, Paris, France; e Service de Neuropédiatrie, Hôpital Necker Enfants Malades, Paris, France

**Keywords:** Mitochondrial myopathy, Heteroplasmy, Muscle biopsy, mtDNA, *MT-TF*

## Abstract

Mitochondrial myopathies are progressive muscle disorders caused by impaired mitochondrial oxidative phosphorylation, leading to reduced adenosine triphosphate production. Skeletal muscles have a high energy demand and are often the first to be affected. In addition to muscular symptoms (muscle weakness, effort intolerance, fatigue), the disease can affect the central and peripheral nervous systems, as well as the heart, liver, kidneys and endocrine system (diabetes). Molecular genetic diagnostic is currently based on leukocyte DNA obtained from blood samples, considered less invasive than muscle biopsy. We report four patients from three families with mitochondrial myopathy associated with ptosis, sensorineural hearing loss, epilepsy, tubulointerstitial nephropathy and cardiomyopathy. Genetic studies identified *MT-TF* variants (m.586G > A, m.601G > A, m.616 T > C) with highly variable heteroplasmy levels in the same patient from one tissue to another (5 % to 70 % mutant load in circulating blood leukocytes and in muscle respectively).

We emphasize the importance of performing mtDNA analysis on muscle DNA, even in patients with negative blood leukocytes mtDNA sequencing, if there is strong clinical suspicion of mitochondrial myopathy.

## 1 Introduction

Mitochondrial myopathies (MM) are progressive muscle disorders with a prevalence of 1/4300 [1], caused by impaired mitochondrial oxidative phosphorylation (OXPHOS), leading to reduced adenosine triphosphate (ATP) production. Skeletal muscles have a high energy demand and are often the first to be affected [2]. In addition to muscular symptoms (muscle weakness, effort intolerance, fatigue), the disease can affect the central and peripheral nervous systems, as well as the heart, liver, kidneys and endocrine system.

MM can be caused by pathogenic variant affecting either mitochondrial or nuclear DNA [3]. The clinical expression of mitochondrial DNA (mtDNA) mutations depends on the percentage of wild type and mutant mtDNA coexisting in any given cell, known as heteroplasmy. Mitochondrial transfer RNA (tRNA) gene mutations are associated with a wide range of pathologies, from isolated myopathy, to multisystem disorders, including encephalopathy, epilepsy, gastrointestinal (GI) dysmotility, hearing loss and life-threatening cardiomyopathy [4]. The diagnostic of MM traditionally relies on specific histological markers in muscle biopsy such as ragged red fibers (RRF) or cytochrome *c* oxidase (COX) negative/succinate dehydrogenase (SDH) positive fibers and measurement of OXPHOS enzymatic activity in muscle. Nowadays, definitive diagnostic is usually based on mtDNA sequencing performed on blood leukocyte DNA [5].

We report three families with MM due to three different pathogenic variants in the mitochondrial *MT-TF* gene, which encodes mitochondrial tRNA^Phe^. In MM, heteroplasmy level can vary significantly in different tissues of a same patient. Clinicians should be aware of this variability to avoid missing a molecular diagnosis of MM.

## 2 Methods

### 2.1 Subjects

The four patients were recruited in two genetic departments (Paris and Bordeaux) belonging to the CARAMMEL French reference network for mitochondrial diseases.

Clinical data including family history, psychomotor development, neurological examination, biochemical and metabolic work-up, and neuroimaging studies were obtained for all patients.

Patient 2 was previously reported [6].

### 2.2 Muscle histopathology

Muscle biopsies from patients 1–3 were frozen in liquid nitrogen. Cryostat sections were used for hematoxylin-eosin-safran (HES), Gömöri trichrome and double COX-SDH enzymatic staining. Myosin heavy chain expression was assessed using monoclonal antibodies against fast and slow isoforms (myosin fast/slow).

### 2.3 OXPHOS spectrophotometric analysis in muscle and heart

OXPHOS spectrophotometric analysis was performed as previously described [7].

### 2.4 Genetic studies

**Fig. 1:**
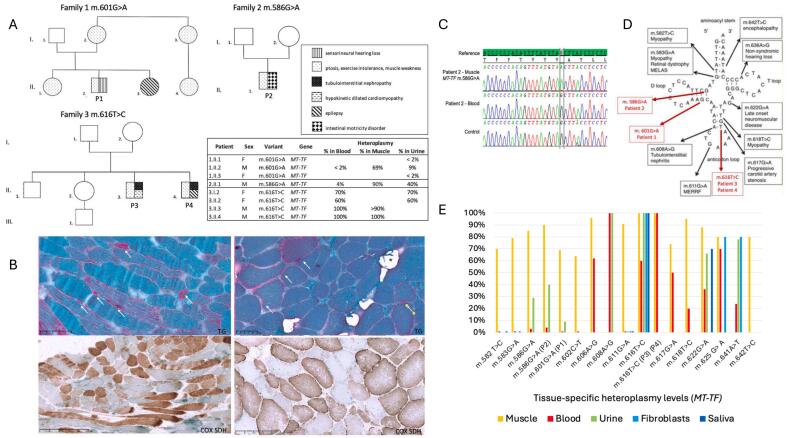
Patients, phenotypes, genetic variants in *MT-TF* gene, mutant loads and segregation of the *MT-TF* variants. A. Families' pedigrees. B. Histological features of muscle from patient 1 (left) and patient 2 (right). Upper panel: Gömöri trichrome staining revealed a typical aspect of mitochondrial myopathy with numerous RRF (white arrows) with large lipid droplets in most fibers (*). Lower panel: Sequential COX-SDH reaction revealed a mosaic pattern of COX-deficiency. C. Sanger sequencing of *MT-TF* showing the m.586G > A substitution in muscle DNA but not in blood leukocytes of P2. D. Schematic diagram of the mitochondrial tRNA^Phe^ cloverleaf structure with the variations reported in this article (in red) and previously reported mutations. Adapted from [10] E. Tissue-specific heteroplasmy levels for each *MT-TF* point mutation reported in the literature.

Total DNA was extracted from peripheral blood, urinary epithelial cells, skeletal muscle cells and saliva using Qiagen or Autopure DNA purification kit and sequenced using next-generation sequencing. Single nucleotide variants (SNVs) were confirmed by Sanger sequencing and segregation analysis was completed in each family (Fig. 1C).

mtDNA was amplified in two fragments by long range PCR (8.2 Kb and 8.3 Kb). Libraries were prepared from 1.5 μg of PCR sheared with a Covaris S2 Ultrasonicator. DNA was sequenced on Illumina MiSeq (Illumina Inc., San Diego, CA) generating 2 × 130 paired-end reads aligned against human mtDNA (NC_ 012920.1) using BWA Burrows-Wheeler Aligner.

### 2.5 Ethics statement

This study adhered to the Declaration of Helsinki and was approved by our institutional review board. Informed consent for diagnostic and research studies was obtained for each patient.

## 3 Results

### 3.1 Case reports

#### 3.1.1 Family 1

Patient 1 (male) was 66 years old at the time of diagnosis. He presented bilateral ptosis, exercise intolerance and sensorineural hearing loss. Metabolic work-up revealed a normal plasma lactate and CPK levels. He had an older sister presenting with ptosis and exercise intolerance and a younger sister with infantile onset epilepsy and muscle fatigability. Their mother, maternal aunt and cousin had ptosis and effort intolerance (Fig. 1A).

#### 3.1.2 Family 2

Patient 2 (male) was 14 years old at the time of diagnosis [6]. He is the only child of unrelated parents with unremarkable family history (Fig. 1A). He was born after a full-term pregnancy, a normal delivery (birth weight 2535 g; height 49.5 cm; head circumference 34 cm) and had normal psychomotor development. At the age of 8, he started to present diffuse myalgias, nocturnal cramps, exercise intolerance after short periods (<5 min), abdominal pain and vomiting following physical exertion. At the last clinical examination at the age of 17, he weighted 34 Kg with a height of 1.67 m (BMI 12.2 kg/m^2^), has reduced muscle mass, diminished strength in the lower limbs (2/5), trunk and limb-girdle weakness, difficulty sitting unaided, and progressive scoliosis. The score obtained in the 6-min walk test was 344 m (normal: 0–900 m), with a high fatigue index of 18 %. Cerebral MRI and cardiac ultrasound were normal. Metabolic workup revealed a high plasma lactate level of 4.8 to 6.9 mmol/L (normal: 0.5 to 1.5 mmol/L) and a high lactate/pyruvate ratio (L/*P* = 61; normal: <20), normal CPK and plasma amino acid chromatography. EMG and motor/sensory nerve conduction were normal.

#### 3.1.3 Family 3

Patient 3 (male) was 28 years old at the time of diagnosis. Family history is not informative, and his parents are not related (Fig. 1A). He was born at term of a regular pregnancy with normal measurements (birth weight 3530 g; height 50 cm; head circumference 35 cm) and had a normal psychomotor development. He presented iron-deficient anemia in infancy.

At age of 4, he presented with pallor, profound asthenia and anorexia, which led to the diagnosis of severe cardiac insufficiency due to dilated cardiomyopathy, hypertension (180/120 mmHg), anemia (8.8 g/dL), renal insufficiency (elevated creatine level 310 μmol/L and urea 38 mmol/L) without proteinuria. He also presented fatigability, exercise intolerance, muscle weakness, and ptosis. Cerebral MRI was normal. A metabolic workup revealed elevated plasma lactate (3.3 mmol/L, normal: 0.5 to 1.5 mmol/L), elevated CSF lactate (2.15 mmol/L; normal: < 2 mmol/L), normal CPK and normal plasma amino acid chromatography. Hypokinetic dilated cardiomyopathy progressed requiring heart transplantation at the age of 15. Tubulointerstitial nephropathy worsened requiring renal transplant at the same age.

Patient 4 (male) is the younger brother of patient 3, aged 25 at the time of diagnosis. He presented tubulointerstitial nephropathy, progressing to hemodialysis-treated end-stage renal failure. He was treated by Lamictal and Rivotril for generalized epileptic seizures following dialysis sessions. Cardiac features include ventricular bigeminy with no other rhythm or conduction disorders, and no cardiac function abnormalities. Neurologically, he complains of fatigability with effort intolerance and muscle weakness associated with ptosis.

### 3.2 Muscle histopathology

Muscle biopsy study of P1 and P2 (Fig. 1B) reveals a typical appearance of mitochondrial myopathy, with numerous ragged red fibers (RRF) shredded by Gömöri trichrome and COX-negative and SDH-positive fibers. P2 biopsy also showed lipid droplets. P3 muscle biopsy did not show significant abnormality apart the low intensity of the COX reaction (not shown). P4 did not have a muscle biopsy.

### 3.3 OXPHOS enzyme activities

Investigation of respiratory chain complex activities showed combined deficiency of complexes I, III and IV in muscle from P2. Complex II and citrate synthase were increased suggesting a compensatory upregulation. An isolated Complex IV defect was detected in muscle and heart of P3.

### 3.4 Molecular analysis of mtDNA

NGS sequencing revealed the presence of a heteroplasmic m.601G > A variant in *MT-TF*, with high mutation load in skeletal muscle of P1 (69 %) while barely detectable in urinary epithelia (9 %) and leukocytes (<2 %). This variant has never been reported so far, is absent from Mitomaster and mtDB databases, and modifies an evolutionarily highly conserved nucleotide. Thus, m.601G > A variant was classified as likely pathogenic. The mutation was <2 % in blood DNA of his two sisters. In P2, a heteroplasmic m.586G > A variation in *MT-TF* was detected in skeletal muscle (>90 % mutant load) and urinary epithelial cells (40 %) while barely detectable mutations were found in circulating leukocytes (4 %). This variation could not be detected in circulating leukocytes and urinary epithelial cells of the mother. This variation affects a phylogenetically conserved nucleotide, located in the D-stem and is listed in the Mitomap database as likely pathogenic. [8]

P3 and P4 presented a m.616 T > C variant in *MT-TF*, that was heteroplasmic (>90 % mutant load) and homoplasmic in blood leukocytes of P3 and P4 respectively. This variant is reported in Mitomap database as likely pathogenic, modifying an evolutionarily highly conserved nucleotide. In asymptomatic mother and sister, this variant was detected in blood (60–70 %) and urine (60–70 %).

## 4 Discussion

We report a novel likely pathogenic *MT-TF* (m.601G > A) variant in a patient presenting MM with exercise intolerance, muscle weakness, fatigability, ptosis, and sensorineural hearing loss. Moreover, we also describe 3 patients carrying two rare *MT-TF* pathogenic variants, m.586G > A and m.616 T > C, only reported in a few patients (Table 1). Pathogenic mutations of tRNAs have been shown to affect global tRNA structure, tRNA processing, modification, aminoacylation, and translation efficiency [9]. The m.586G > A and m.601G > A variants, located in the D branch, could affect tRNA stability, as previously demonstrated for the m.582 T > C and m.583G > A variants [9,10]. The m.616 T > C variant modifies an evolutionarily conserved nucleotide, the only pseudo-uridine in mitochondrial tRNA^Phe^, and disrupts the last nucleotide pairing before the anticodon loop (Fig. 1D).

**Table 1:** Overview of clinical features of individuals carrying variants in *MT-TF* and tissue-specific mutant load reported in MitoMap.

Variant	Reference	Phenotype	Age at onset (yrs)	Age at diagnosis (yrs)	Pathogenicity	Mutant load					Heredity
						Muscle	Blood	Urine	Fibroblasts	Saliva	
m.582 T > C	Moslemi et al. 2004	Mitochondrial myopathy, ptosis	50	70	Possibly pathogenic	70 %	0 %	ND	ND	0 %	de novo
m.583G > A	Hanna et al. 1998	MELAS	12	32		58 %	0 %	ND	0 %	ND	de novo
m.583G > A	Darin et al. 2006	Mitochondrial myopathy, retinopathy	14	17	Pathogenic	79 %	0 %	ND	0 %	ND	de novo
m.586G > A	Young et al. 2010	Mitochondrial myopathy, encephalopathy, lactic acidosis, gastrointestinal dysmotility, Tubulointerstitial nephropathy	45	57	Likely pathogenic	85 %	3 %	29 %	ND	ND	maternal
m.586G > A	D'Aco et al. 2013	Encephalopathy, regression, lactic acidosis, gastrointestinal dysmotility, Tubulointerstitial nephropathy	1			heteroplasmic	heteroplasmic	ND	ND	ND	de novo
m.586G > A	Barcia et al. 2019	**Patient 2**	8	14	Likely pathogenic	>90 %	4 %	40 %	ND	ND	de novo
m.591C > T	Viering et al. 2022	Tubulointerstitial nephropathy, muscle weakness, epilepsy			Likely pathogenic	ND	98 %	ND	ND	ND	
m.601G > A	This report	**Patient 1**		66	Likely pathogenic	69 %	0 %	9 %	ND	ND	
m.602C > T	Sakiyama et al. 2011	Mitochondrial myopathy, ptosis, dysarthria, cataract, SNHL	63	73	Likely pathogenic	64 %	0 %	ND	ND	ND	maternal
m.606 A > G	Kleinle et al. 1998	Rhabdomyolysis, myoglobinuria	15	30		96 %	62 %	ND	ND	ND	maternal
m.608 A > G	Tzen et al. 2001	MELAS, epilepsy Tubulointerstitial nephropathy	0	1	Likely pathogenic	ND	100 %	100 %	ND	ND	maternal
m.610 T > C	Indelicato et al. 2023	Myoclonic epilepsy Developmental regression NORSE	7 29			ND ND	97 % 97 %	ND ND	100 % ND	ND ND	maternal
m.611G > A	Mancuso et al. 2004	MERRF	20	42	Likely pathogenic	91 %	0 %	0 %	0 %	0 %	de novo
m.616 T > C	Viering et al. 2022	Tubulointerstitial nephropathy, muscle weakness, epilepsy				ND	100 %	ND	ND	ND	ND
m.616 T > C	Connor et al., 2017	Tubulointerstitial nephropathy muscle weakness, epilepsy				ND	100 %	ND	ND	ND	ND
m.616 T > C	Zsurka et al. 2010	Epileptic encephalopathy, Tubulointerstitial nephropathy	1	17	Pathogenic	100 %	100 %	100 %	99 %	ND	maternal
m.616 T > C	Lorenz et al. 2020.	Epileptic encephalopathy, Tubulointerstitial nephropathy	3	5		ND	ND	ND	ND	ND	ND
m.616 T > C	Xu et al. 2022	Tubulointerstitial nephropathy, muscle weakness, epilepsy				ND	>90 %	100 %	100 %	100 %	ND
m.616 T > C	This report	**Patient 3**	4	28	Pathogenic	ND	>90 %	ND	ND	ND	maternal
m.616 T > C	This report	**Patient 4**		25	Pathogenic	ND	100 %	ND	ND	ND	maternal
m.616 T > G	Zsurka et al. 2010	Myoclonic epilepsy, Tubulointerstitial nephropathy		15	Likely pathogenic	100 %	100 %	ND	ND	ND	ND
m.617G > A	Iizuka et al. 2009	MELAS, carotid artery stenosis	8	40	Likely pathogenic	74 %	50 %	ND	ND	ND	maternal
m.618 T > C	Kleinle et al. 1998	Mitochondrial myopathy	26	36	Possibly pathogenic	95 %	20 %	ND	ND	ND	maternal
m.618 T > G	Yarham et al. 2011	Mitochondrial myopathy, PEO, ptosis, dysphagia		25	Likely pathogenic	76 %	0 %	0 %	ND	ND	de novo
m.622G > A	Deschauer et al. 2006	Exercice intolerance, SNHL	62	66		88 %	36 %	66 %	ND	70 %	maternal
m.625 G > A	Sudo et al. 2011	Epilepsy, SNHL	6	11	Likely pathogenic	80 %	70 %	ND	80 %	ND	ND
m.628C > T	Dowlati et al. 2013	SNHL	2	7		ND	ND	ND	ND	ND	maternal
m.636 A > G	Konings et al. 2008	SNHL		1		ND	ND	ND	ND	ND	ND
m.641 A > T	Itkis et al. 2019	Myoclonic epilepsy, motor regression with spastic tetraplegia	8		Possibly pathogenic	ND	24 %	78 %	80 %	ND	ND
m.642 T > C	Valente et al. 2009	Mitochondrial myopathy, encephalopathy, PEO, ptosis, SNHL, peripheral neuropathy, gait ataxia		47	Possibly pathogenic	80 %	ND	ND	ND	ND	ND
m.643 A > G	Viering et al. 2022	Tubulointerstitial nephropathy, muscle weakness, epilepsy			VUS	ND	100 %	ND	ND	ND	ND

List of abbreviations. MELAS: Mitochondrial Encephalomyopathy, Lactic Acidosis and Stroke-like episodes; MERRF: Myoclonic Epilepsy with Ragged Red Fibers; ND: not determined; NORSE: New-Onset Refractory Myoclonic Status Epilepticus; PEO: Progressive External Ophthalmoplegia; SNHL: Sensorineural Hearing Loss; VUS: Variants of Unknown Significance.

Patients with mitochondrial tRNA mutations have complex clinical symptoms with highly heterogeneous phenotype. *MT-TF* pathogenic variants have been associated to a variety of clinical symptoms, including myopathy, ptosis, sensorineural hearing loss, epilepsy and tubulointerstitial nephropathy. The age of onset of symptoms and their severity is variable, as is often the case with mitochondrial diseases (Table 1).

A previous report described a 16-month-old girl with the *MT-TF* m.586G > A variant (heteroplasmic in muscle and blood) associated with failure-to-thrive, developmental regression, persistent lactic acidosis, hypotonia, GI dysmotility and renal impairment with chronic tubulointerstitial fibrosis, progressing to end-stage renal disease [11]. This *MT-TF* m.586G > A variant has also been reported in a 57 yrs-old woman with progressive neurodegenerative disorder, akinesia-rigidity, abnormal movements, dementia, and psychiatric disorder [12]. This patient also had lactic acidosis, GI dysmotility, renal failure. No MM was observed but muscle biopsy showed markers of MM. Heteroplasmy level was 3 % in blood and 85 % in muscle, comparable with P2 heteroplasmy level for this specific m.586G > A variant.

The m.616 T > C variant has been described in patients presenting epilepsy and tubulointerstitial kidney disease. Muscle weakness was often reported without further investigation. Analysis of muscle histology when performed showed decreased overall COX staining [13].

Our report aims to emphasize that MM associated with mitochondrial tRNA^Phe^ mutations are not uncommon and are probably underdiagnosed because of the highly variable mutant load in different tissues. Indeed, genetic analyses are most often performed on leukocyte DNA as muscle biopsy remains an invasive procedure. High-throughput sequencing of mtDNA, with a coverage of >1000 reads of each nucleotide, can detect a low load of mutant mtDNA allowing genetic diagnosis. However, in P1 and P2, the mutation was not detectable in leukocytes, which has also been reported for other *MT-TF* mutations with a mutant load less than 2 % [4,6,9,10,[13], [14], [15], [16], [17], [18], [19]]. Similarly, certain variants of the *MT-TF* gene have been investigated in several tissues to assess the variability of heteroplasmy levels (Fig. 1E). Therefore, a strong clinical suspicion of MM should prompt to test additional tissues, especially muscle, even if no mtDNA variant could be detected in leukocyte. Samples of urine or saliva are readily available to assess heteroplasmic mtDNA variations and are easier to collect than muscle or fibroblasts. Thus, the presence of 40 % mutated molecules in the urine of P2 reinforces the diagnostic hypothesis.

It seems essential to continue the precise clinical and histological study of mitochondrial tRNA variants, in conjunction with existing molecular databases to confirm diagnosis.

## CRediT authorship contribution statement

**Sylvia Rose:** Conceptualization, Writing – original draft, Writing – review & editing. **Aurélien Trimouille:** Conceptualization, Supervision, Validation. **Didier Lacombe:** Conceptualization, Supervision, Validation. **Edoardo Malfatti:** Supervision, Validation. **Zahra Assouline:** Supervision, Validation. **Julie Steffann:** Supervision, Validation. **Isabelle Desguerre:** Supervision, Validation. **Arnold Munnich:** Supervision, Validation. **Agnès Rötig:** Conceptualization, Supervision, Validation, Writing – review & editing. **Giulia Barcia:** Conceptualization, Supervision, Validation, Writing – review & editing.

## Declaration of competing interest

None.

## Data Availability

No data was used for the research described in the article.
